# Structures of Reaction Products and Degradation Pathways of Aflatoxin B_1_ by Ultrasound Treatment

**DOI:** 10.3390/toxins11090526

**Published:** 2019-09-12

**Authors:** Yuanfang Liu, Mengmeng Li, Yuanxiao Liu, Ke Bian

**Affiliations:** 1 College of Food Science and Technology, Henan University of Technology, Zhengzhou 450001, China; 2 School of Chemistry and Chemical Engineering, Zhengzhou Normal University, Zhengzhou 450044, China

**Keywords:** reaction products, aflatoxin B_1_, UHPLC-Orbitrap-MS, ultrasound treatment

## Abstract

Ultrasound is an emerging decontamination technology with potential use in the global food processing industry. In the present study, we explored power ultrasound for processing aqueous aflatoxin B_1_ (AFB_1_). AFB_1_ was degraded by 85.1% after 80 min of ultrasound exposure. The reaction products of AFB_1_ were identified and their molecular formulae elucidated by ultra-high-performance liquid chromatography Q-Orbitrap mass spectrometry. Eight main reaction products were found, and their structures were clarified by parental ion fragmentation. Two degradation pathways were proposed according to the degradation product structures: One involved the addition of H• and OH• radicals, whereas the other involved H_2_O_2_ epoxidation and H•, OH•, and H_2_O_2_ oxidation of AFB_1_. Ultrasound treatment significantly reduced AFB_1_ bioactivity and toxicity by disrupting the C8=C9 double bond in the furan ring and modifying the lactone ring and methoxy group.

## 1. Introduction

Aflatoxins are the most common mycotoxins. They are secondary metabolites of *Aspergillus flavus* that can reduce food quality [1] and have adverse health effects [2,3]. Aflatoxin B_1_ (AFB_1_) is the strongest teratogen, mutagen, and hepatocarcinogen known. The International Agency for Research on Cancer (IARC) has rated AFB_1_ as a class 1 carcinogen [4]. AFB_1_ is distributed mainly in maize, peanut, rice, wheat, and other crops, as well as in their oil-based by-products. Small quantities of AFB_1_ are also found in dairy products and condiments [5].

Prevention of mycotoxin contamination is the most economically effective way of reducing the risks posed by aflatoxin exposure. However, additional processing is often insufficient for the decontamination and detoxification of food and feed products. Detoxification is important in making aflatoxin-contaminated grains usable, and thus, safeguarding the food industry. Over the last several decades, physical, biological, and chemical strategies for aflatoxin degradation and their effects on aflatoxin content have been extensively investigated [6,7,8]. Detoxification treatments include electron beam irradiation [9], citric and lactic acids [10], ozone gas [11], cold plasma [12], and neutral electrolytic water [13]. However, most methods have disadvantages, such as nutrient loss, inconvenience of operation, reduction of sensory attributes, and high costs. Consequently, these techniques are of little practical use. Thus, there is a high demand for effective, specific, and environment-friendly technologies in this regard.

Ultrasound is emerging as an environment protection method, as it produces no secondary pollutants [14]. Cavitation bubbles in liquid media are generated by ultrasound when acoustic wave occurs during the rarefaction cycle. The bubbles continue to expand until they collapse after reaching a critical radius [15]. When cavitation bubbles collapse, they raise the temperature to >5000 °C and the pressure to >1000 atm [16]. Under these extreme conditions, contaminant compounds in the vicinity are degraded. The covalent bonds in water are broken, and OH• radicals are formed. These radicals oxidize aqueous contaminants [15]. Ultrasound treatment has received increasing attention as a means of degrading various micropollutants, such as parathion [17], 5-methylbenzotriazole [18], ibuprofen [19], and ethyl paraben [20]. To the best of our knowledge, however, there has been no extensive study on the reaction products of AFB_1_ treated with ultrasound or their potential toxicity. Thus, little is known about the reaction mechanisms involved in the degradation of AFB_1_ by ultrasound treatment, and elucidation of the ultrasound process is the basis for the development of its future applications. This study is a continuation of a previous investigation of the treatment of mycotoxins (AFB_1_, deoxynivalenol, zearalenone, and ochratoxin A) with ultrasound [21]. The goals of this study were to identify the molecular structures of the reaction products of AFB_1_, elucidate the decomposition mechanisms and reaction pathways of AFB_1_, and determine the particular factors that lead to AFB_1_ degradation. Toxicity of the reaction products was also correlated with their structures.

## 2. Results and Discussion

### 2.1. Formation of AFB_1_ Reaction Products as A Function of Ultrasound Treatment Time

**Figure 1:**
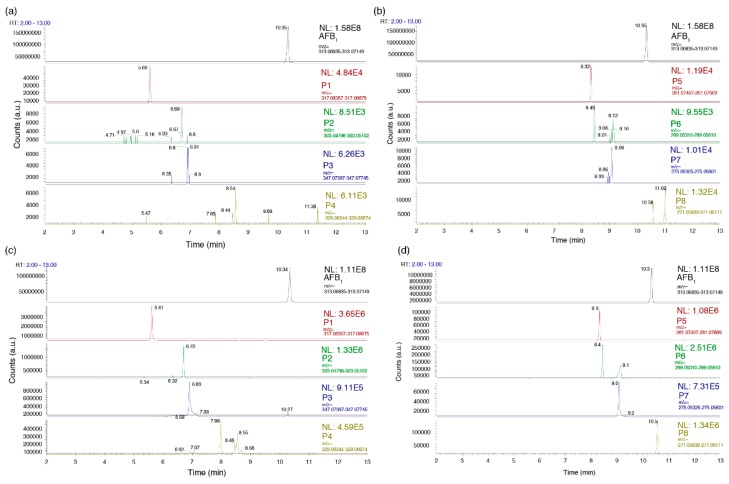
Total-ion chromatograms of untreated Aflatoxin B_1_ (AFB_1_) (10 μg·mL^−1^) in ultrapure water (**a**,**b**) and AFB_1_ in ultrapure water exposed to ultrasound for 40 min (**c**,**d**).

Chromatography/mass spectrometry data for AFB_1_ before and after ultrasound treatment were collected by UHPLC-Q-Orbitrap high-resolution mass spectrometry (Orbitrap MS–MS). MS/MS fragment ion data were collected simultaneously. The original data were imported into SIEVE v. 2.0 (Thermo Fisher Scientific, Bremen, Germany) for differential expression analysis. The software identified eight new products generated after 40 min of US treatment of AFB_1_, which was mainly degraded; these were labeled P1–P8 (Figure 1). Their signal-to-noise ratios were below the detection threshold in the blank experiment. Figure 1C,D shows that the retention times and peak shapes of the eight reaction products were satisfactorily separated. Their response values were sufficiently high for detection. The compounds were found in the samples after 30, 40, 60, and 80 min of ultrasound treatment.

**Figure 2:**
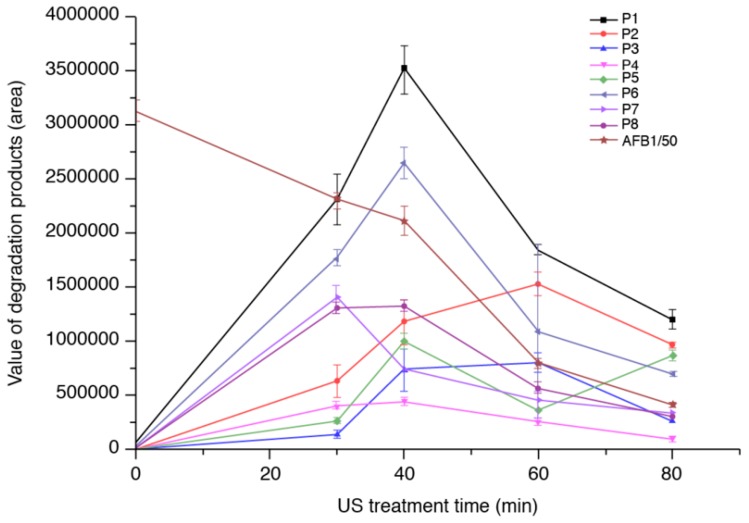
Relative change in the responses of AFB_1_ and its reaction products (P1–P8) in water with increasing ultrasound (US) treatment time. (Note: The AFB_1_ values were normalized so they could be displayed in the same scale.).

Figure 2 shows the changes in the responses of AFB_1_ and its reaction products (P1–P8) in water with increasing US treatment time. AFB_1_ gradually decomposed with increasing treatment time. The levels of all reaction products except P2 and P7 gradually increased within the first 40 min of US treatment and decreased thereafter. The observed decreases in the levels of certain degradation product suggest that these substances may have been reaction intermediates subsequently converted to other reaction products. There was a tremendous decay trend after the observed decrease in AFB_1_ level during the 40-min ultrasound treatment. This finding is in line with our previous findings for aflatoxin B_1_ subjected to ultrasound treatment [21]. The areas of the peaks indicated that ~85.1% of the AFB_1_ was degraded after 80 min of ultrasound treatment.

### 2.2. Molecular Formulae of the AFB_1_ Reaction Products

To help identify the molecular formulae of the reaction products of AFB_1_, their retention times, proposed formulae, experimental masses, mass errors, index of hydrogen deficiency (IHD), and score data, as well as those for AFB_1_, are summarized in Table 1. The overall score ranged from 0–100%. Scores closer to 100% were preferable. Relative to the ideal mass gained from the hypothetical molecular formula, the mass measured by the Q-Orbitrap-MS experiments had an error <0.5 mmu.

As accurate masses of these eight reaction products were generated by SIEVE v. 2.0 (Thermo Fisher Scientific, Waltham, MA, USA), their elemental compositions could be speculated by considering all possible permutations. AFB_1_ was processed by ultrasound treatment in pure water. Thus, the reaction products of AFB_1_ should only be formed of hydrogen, carbon, and oxygen. The molecular formulae were predicted by Xcalibur v. 3.0 (Thermo Fisher Scientific) and according to exact masses of the compounds. For example, some possible molecular compositions of P-1 were C_16_H_13_O_7_, C_12_H_13_O_10_, and C_17_H_17_O_6_ with scores of 89.1, 85.1, and 80.4%, respectively. As C_16_H_13_O_7_ showed a higher score than the others, it is most possibly the correct molecular formula for P-1. The IHD of the reaction products of AFB_1_ should be closer to that of AFB_1_. The IHD of AFB_1_ is 11.5, and there are 17 carbon atoms and 12 hydrogen atoms in one AFB_1_ molecule. The IHDs of the molecular formulae C_16_H_13_O_7_, C_12_H_13_O_10_, and C_17_H_17_O_6_ are 10.5, 6.5, and 9.5, respectively. As the IHD of C_16_H_13_O_7_ most nearly approaches that of AFB_1_, it is the most likely molecular formula for P-1.

### 2.3. Proposed Structures of the AFB_1_ Reaction Products

**Figure 3:**
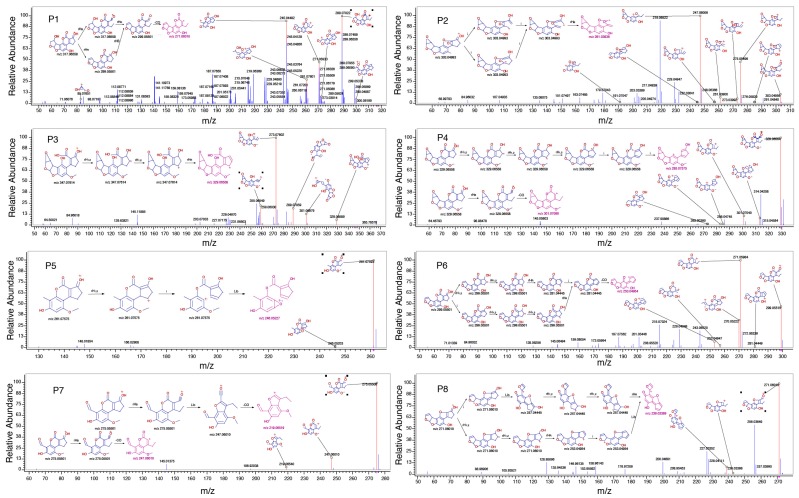
Orbitrap MS–MS spectra and possible fragmentation (insets) of the reaction products of AFB_1_ upon ultrasound treatment.

**Figure 4:**
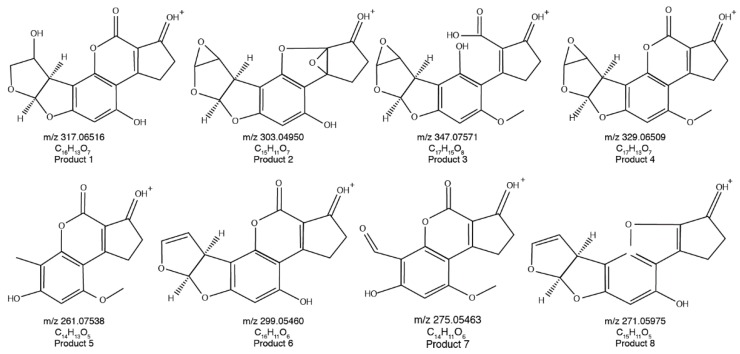
Proposed structures of the reaction products (P1–P8) of AFB_1_ generated upon ultrasound treatment.

To elucidate the structures of the eight reaction products of AFB_1_, the exact masses of their fragmentation ions were evaluated by Orbitrap MS–MS. In this way, the most probable structures of the reaction products of AFB_1_ and their parent compounds could be determined. Based on the parent ions’ masses and fragments gained from MS–MS, the structures of the eight reaction products are displayed in Figure 3. The structures of the AFB_1_ reaction products, generated by US treatment, are shown in Figure 4. The structures of the eight reaction products (P1–P8) resemble that of AFB_1_. US treatment modified the AFB_1_ furofuran ring (P-1–P-5 and P-7), lactone ring (P-2, P-3, and P-8), and methoxy group (P-1, P-2, P-6, and P-8).

### 2.4. Degradation Mechanism and Reaction Pathway of AFB_1_ upon US Treatment

High-power ultrasound treatment modifies the physicochemical properties of food-borne pathogens during processing [22]. The ultrasound treatment causes cavitation in which the covalent bonds of water molecules are broken, and numerous free radicals are generated that oxidize contaminants in water [15]. The temperature of the sample treated with ultrasound is ~60 °C, which is below the temperature required for thermal degradation of AFB_1_ in water (120 °C) [23]. The heat generated during ultrasound treatment has a negligible effect on AFB_1_ degradation. We believe that the free radicals, generated during ultrasound treatment, lead to the degradation of AFB_1_. Furthermore, it should be noted that the temperature rises by cavitation, as discussed previously, occurs solely in the mini bubbles generated; however, the temperature change, in this case, is stable, continuous, and uniform.

(1) $$H_{2}O\overset{\mathrm{Sonication}}{\to }H+\mathrm{OH}$$

(2) $$\mathrm{OH}+\mathrm{OH}\to H_{2}O_{2}$$

(3) OH+Mycotoxins→Degradation Products

(4) $$H_{2}O_{2}\;\overset{\mathrm{Sonication}}{\to }2\mathrm{OH}$$

(5) $$\mathrm{OH}+H_{2}O\to H_{2}O_{2}+H$$

Ultrasound treatment generates numerous hydroxyl radicals (Reaction 1). At low mycotoxin concentrations, the hydroxyl radicals combine to form hydrogen peroxide (Reaction 2). At suitably high AFB_1_ concentrations, the hydroxyl radicals degrade the mycotoxin molecules (Reaction 3). Sonolysis of water yields free radicals, such as hydrogen atoms and hydroxyl, as well as hydrogen peroxide [24,25]. Under ultrasound treatment with cavitation, the water molecules are broken into free radicals, which degrade various micropollutants, such as parathion [17], 5-methylbenzotriazole [18], ibuprofen [19], and ethyl paraben [20]. Sonolysis generates highly reactive hydroxyl radicals, which recombine outside of the bubbles even at very low scavenger concentrations to form hydrogen peroxide that is released into the medium [25]. The hydroxyl radicals may also attack AFB_1_ and initiate its degradation. Aflatoxins are also effectively degraded by aqueous ozone because it too generates hydroxyl radicals [26,27]. The AFB_1_ reaction products P-1 (C_16_H_13_O_7_) and P-3 (C_17_H_15_O_8_) are the major by-products of AFB_1_ treatment with aqueous ozone [26,28]. The AFB_1_ reaction products P-4 (C_17_H_13_O_7_), P-5 (C_14_H_13_O_5_), and P-7 (C_14_H_11_O_6_) are the reaction products of AFB_1_ treated with high-voltage atmospheric cold plasma [29]. In this study, we identified additional major reaction products of AFB_1_. Thus, degradation of AFB_1_ by ultrasound treatment sheds light on new pathways.

**Figure 5:**
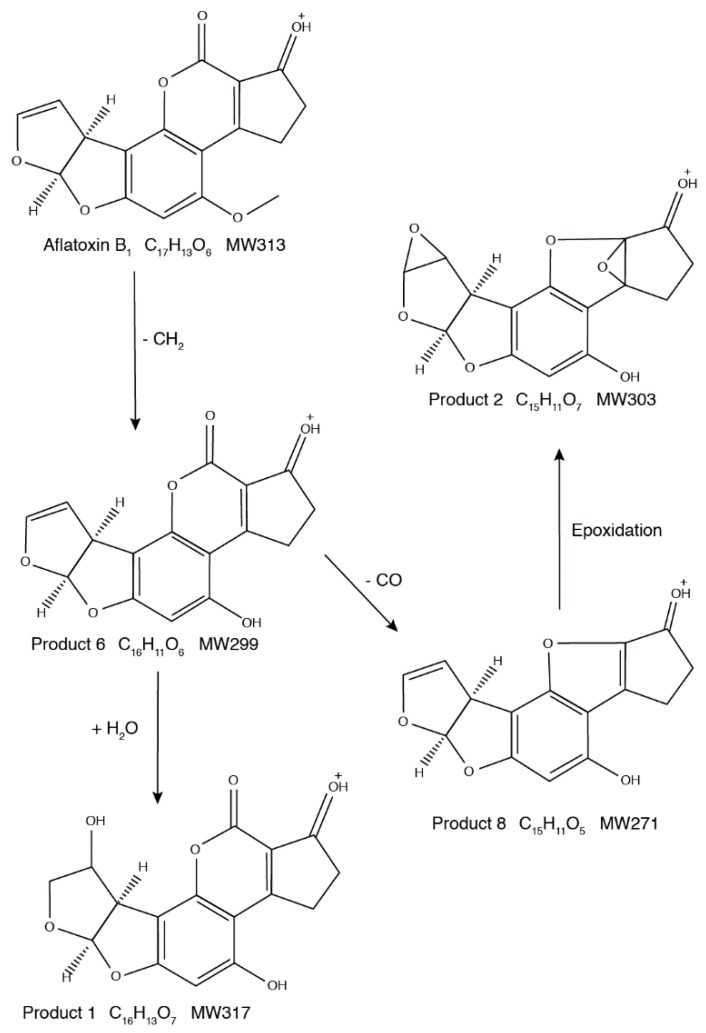
First degradation pathway of AFB_1_ under ultrasound treatment.

**Figure 6:**
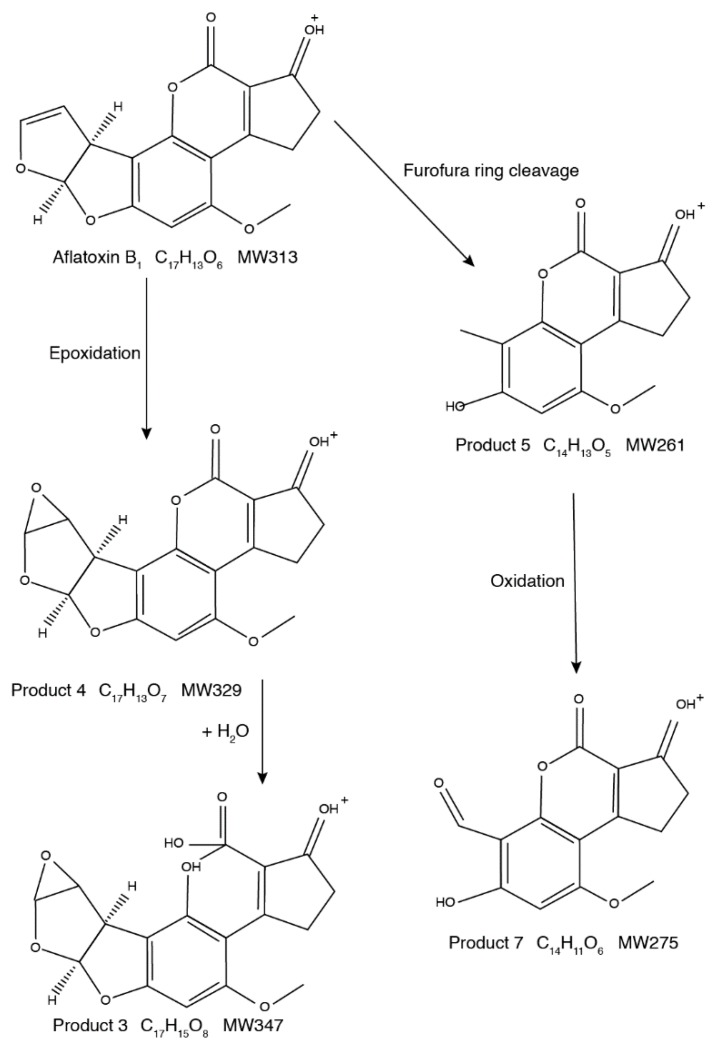
Second degradation pathway of AFB_1_ under ultrasound treatment.

Based on the structures of the eight AFB_1_ reaction products generated upon ultrasound treatment, two degradation pathways were proposed (Figure 5 and Figure 6). In the first, AFB_1_ is degraded to C_16_H_13_O_7_ (*m*/*z* 317.06516), C_15_H_11_O_7_ (*m*/*z* 303.04950), C_16_H_11_O_6_ (*m*/*z* 299.05460), and C_15_H_11_O_5_ (*m*/*z* 271.05975). In the second, AFB_1_ is degraded to C_17_H_15_O_8_ (*m*/*z* 347.07571), C_17_H_13_O_7_ (*m*/*z* 329.06509), C_14_H_13_O_5_ (*m*/*z* 261.07538), and C_14_H_11_O_6_ (*m*/*z* 275.05463).

The first pathway involves mainly the loss of methyl and methanol groups, additions, and epoxidations. The first step is the loss of the methyl residue on the methoxy group on the benzene side chain to form C_16_H_11_O_6_ (*m*/*z* 299.05460). The next reaction has two branches. In the first, the C8=C9 double bond of AFB_1_ is hydrated to produce C_16_H_13_O_7_ (*m*/*z* 317.06516). In the second, methanol is lost from the lactone ring to generate C_15_H_11_O_5_ (*m*/*z* 271.05975). Epoxidation of the double bonds in C_15_H_11_O_5_ leads to the formation of C_15_H_11_O_7_ (*m*/*z* 303.04950). Based on the first pathway of AFB_1_ degradation, the essential factors are the hydrogen atom (H•), hydroxyl radical (OH•), and hydrogen peroxide. These molecules originated from water molecules that were broken down by the ultrasound treatment [24]. The hydrogen atom and the hydroxyl radical form new reaction products by hydration and hydrogenation. Hydrogen peroxide, which is also generated by ultrasound treatment, contributes to epoxidations.

The second pathway involves mainly epoxidations, oxidations, and additions. The left branch leads to the formation of C_17_H_13_O_7_ (*m*/*z* 329.06509) through epoxidation of the C8=C9 double bond of AFB_1_. Epoxidation is driven by the hydrogen peroxide generated during ultrasound treatment. Hydrogen peroxide reacts with double bonds and leads to the formation of epoxides [29,30]. Adding water molecules to the double bond at the lactone ring (hydration) produces C_17_H_15_O_8_ (*m*/*z* 347.07571). In the other branch, the furofuran ring in the AFB_1_ molecule is broken down and C_14_H_13_O_5_ (m/z 261.07538) is generated. Further oxidation of this by-product contributes to the formation of C_14_H_11_O_6_ (*m*/*z* 275.05463).

Ultrasonic waves can break down oxygen gas molecules dissolved in water [31]. Several studies have shown that ultrasound bombardment of water generates hydroxyl (OH•), hydrogen atoms (H•), and hydrogen peroxide [32,33]. As the concentrations of these species were not quantified during ultrasound treatment, it is unknown which species reacted with AFB_1_. Probably, the degradation of AFB_1_ occurs through uniting all these species, because they coexist during the ultrasound process and are interconvertible. In summary, the second degradation pathway involves epoxidation by H_2_O_2_ and oxidation through the combined effects of H•, OH•, and H_2_O_2_.

### 2.5. Toxicity of the Reaction Products

The toxicity of aflatoxins has been extensively investigated since their discovery in the early 1960s [34,35,36]. Structure-bioactivity relationships of aflatoxins have also been analyzed [37,38]. The molecular structure of AFB_1_ is conducive to causing severe toxicity, mutagenicity, and carcinogenicity. Changes in the furofuran or lactone rings or the cyclopentenone or methoxy moieties would markedly reduce the biological activity of AFB_1_ [38]. In the present study, IHD was calculated based on the data obtained by Q-Orbitrap and indicated the number of double bonds and rings in the molecule. The IHD of AFB_1_ is 11.5. Table 1 shows that the IHDs of 75% of the AFB_1_ reaction products were lower than 11.5. As certain reaction products had IHD = 9.5 and others had IHD = 10.5, double bond additions were thought to have occurred. There are two sites on the AFB_1_ molecule essential for its toxicity: One is located at the C8=C9 double bond of the furan ring wherein aflatoxin-DNA, and aflatoxin-protein interactions occur; the other is situated at the lactone ring. It is shown in Figure 4 that six of the eight proposed major AFB_1_ reaction products showed changes in their double bonds and were very different from AFB_1_ via further modifications of the furofuran ring (P-1–P-5 and P-7), lactone ring (P-2, P-3, and P-8), and methoxy group (P-1, P-2, P-6, and P-8). Based on the structure-bioactivity relationships, we believe that the toxicity of the products of ultrasound-treated AFB_1_ will be markedly lower than that of AFB_1_ itself.

The aforementioned findings were confirmed by an earlier study on AFB_1_ treated with aqueous ozone. In those papers, the toxicity of the AFB_1_ reaction products was substantially lower or even negligible relative to that of AFB_1_ itself [39,40]. Nevertheless, we recommend that additional bioactivity tests, such as duckling and Ames, or cell model studies be conducted to verify that ultrasound-treated AFB_1_ samples are safe for animals and humans.

The reaction products generated from pure AFB_1_ exposed to ultrasound are complex. In the present study, ultra-high-performance liquid chromatography Q-Orbitrap mass spectrometry (UHPLC-Orbitrap-MS) proved to be the most suitable tool for the elucidation of these breakdown products of AFB_1_. Accurate mass measurements by Orbitrap-MS clarified the elemental composition of the ions (molecules and fragments), while SIEVE v. 2.0 and Mass Frontier v. 7.0 furnished complementary structural information. In the present study, the structures of eight key reaction products of AFB_1_ and two possible reaction pathways were proposed. The structures of these by-products suggest that free radical participate in AFB_1_ degradation. A toxicity assessment of these reaction products has also been offered. As additional reactions occurred in the formation of most of the reaction products, the toxicity of these compounds were considerably lower than that of AFB_1_.

The findings of the present study present pulsed ultrasound treatment as a promising method to degrade AFB_1_ under specific conditions, including in aqueous solutions. In the future, we would like to extend our research to gain insights into how the ultrasound technology works in a food matrix, such as corn or peanuts.

## 3. Materials and Methods

### 3.1. Chemicals and Reagents

Aflatoxin B_1_ (purity > 98%) was purchased from J&K Chemical Ltd. (Shanghai, China). Aflatoxin B_1_ stock standard solution (100 mg·L^−1^) was prepared by weighing out exactly 5 mg AFB_1_ powder and dissolving it in 50 mL MS-grade acetonitrile. The AFB_1_ stock standard solution was stored in a freezer at −18 °C before experimental use. Aflatoxin B_1_ sample solution (10 mg·L^−1^) was prepared by evaporating 2.5-mL aliquots of the standard stock solution and re-dissolving them in 25 mL ultrapure water. LC-MS-grade methanol and acetonitrile were purchased from Thermo Fisher Scientific. Formic acid (purity > 98%) was obtained from Panera (Barcelona, Spain). Water (resistivity = 18.2 MΩ·cm, 25 °C) was produced by an ultrapure purification system (EMD Millipore, Billerica, MA, USA).

### 3.2. AFB_1_ Treatment with Power Ultrasound

Each 25-mL Aaflatoxin B_1_ sample solution was placed in a 50-mL beaker. The probe of the ultrasonic processor was immersed into the sample solutions to a depth of 1.5 cm, and vibration was initiated for the ultrasound experiments. A 550-W power ultrasonic instrument (Branson Ultrasonic Co., Shanghai, China) with a 13-mm probe was used for the ultrasound treatment. The frequency was a constant 20 kHz. The energy input was calculated as the average power per unit volume of the water samples (W·cm^−3^). Several preliminary trials were run to determine the most suitable power intensity; for AFB_1_ degradation, this was determined to be 6.6 W·cm^−3^. The treatment was carried out in pulsed mode. The AFB_1_ samples were treated for 30, 40, 60, or 80 min at a power intensity of 6.6 W·cm^−3^ and the treatments were conducted in triplicate. The processed sample solutions were transferred to 25-mL volumetric flasks. To compensate for evaporation during the ultrasonic treatment, ultrapure water was added to certain volumetric flasks to restore the sample solutions to their original 25 mL.

### 3.3. UHPLC–MS Analysis

Ultrasound-treated and untreated AFB_1_ samples in ultrapure water were carefully evaporated, re-dissolved in methanol:water (25:75), vortexed for 1 min, and centrifuged at 4 °C for 10 min at 12,000× *g*. One milliliter of the supernatant was then placed into the UHPLC-MS/MS system. The UHPLC-MS/MS system was supplied by Thermo Fisher Scientific. The chromatographic instrument was fitted with an Acquity C18 column (100 mm × 2.1 mm) with 1.7-μm particle size. The Q-Orbitrap mass spectrometer was an MS/MS detector.

Chromatographic analyses were performed by gradient elution. Eluent A was acetonitrile with 0.1% formic acid, and eluent B was an aqueous solution of 0.1% formic acid. Gradient elution began with 10% A for 1 min, which was linearly increased up to 95% in 20.0 min. Then, this status was held for another 1.0 min, returned to 10% eluent A in 1.0 min, and re-equilibrated for another 7.0 min. The flow rate was 0.30 mL·min^−1^, and the column temperature was maintained at 35 °C. Ten microliters aliquots of the sample extract were placed into the chromatographic system. The MS was operated in positive electrospray ionization (ESI) mode, and the data were obtained within 50−1100 *m*/*z*.

### 3.4. UHPLC-MS/MS Analysis

The operational parameters of mass spectrometry were a spray voltage of 3.0 kV, heater temperature of 350 °C, and a capillary temperature of 250 °C. The flow rates of the sheath and auxiliary gases were 25 and 5 arb. units, respectively. Dissociation was induced using Ar as the collision gas in the collision cell. High mass accuracy fragmentation data were collected in the data-dependent scanning mode. Data were gained in a full-scan analysis with a resolution of 70,000. The original spectral data were collected and analyzed with Xcalibur v. 3.0, the reaction products were screened with SIEVE v. 2.0, and Mass Frontier v. 7.0 was used to predict the degradation path (all from Thermo Fisher Scientific).

### 3.5. Statistical Analyses

All data were compared using analysis of variance (ANOVA). When the data were considered statistically significant, differences between means were determined using Duncan’s multiple range post hoc tests (*p* < 0.05). The statistical analyses were calculated using Statistical Product and Service Solutions (SPSS, 2010) software (IBM, Amund City, NY, USA).

## Figures and Tables

**Table 1:** Hypothetical formulae for the AFB_1_ reaction products.

Proposed Product	Retention Time (min)	Hypothetical Formula	Determined Mass (*m*/*z*) ^1^	Error (mmu)	IHD ^2^	Score (%)
1	5.69	C_16_H_13_O_7_	317.06516	−0.419	10.5	89.1
2	6.82	C_15_H_11_O_7_	303.04950	−0.429	10.5	87.6
3	6.99	C_17_H_15_O_8_	347.07571	−0.434	10.5	88.9
4	7.17	C_17_H_13_O_7_	329.06509	−0.487	11.5	92.5
5	8.42	C_14_H_13_O_5_	261.07538	−0.370	8.5	88.3
6	8.54	C_16_H_11_O_6_	299.05460	−0.415	11.5	90.9
7	9.21	C_14_H_11_O_6_	275.05463	−0.385	9.5	82.5
8	10.68	C_15_H_11_O_5_	271.05975	−0.350	10.5	85.2
AFB_1_	10.45	C_17_H_12_O_7_	313.07025	−0.415	11.5	99.2

^1^*m*/*z* of [M + H]^+^. ^2^ IHD: Index of hydrogen deficiency.
